# Behavioral and neurocognitive effects of judo training on working memory capacity in children with ADHD: A randomized controlled trial

**DOI:** 10.1016/j.nicl.2022.103156

**Published:** 2022-08-17

**Authors:** Sebastian Ludyga, Manuel Mücke, Rahel Leuenberger, Fabienne Bruggisser, Uwe Pühse, Markus Gerber, Andrea Capone-Mori, Clemens Keutler, Mark Brotzmann, Peter Weber

**Affiliations:** a University of Basel, Department of Sport, Exercise and Health, Basel, Switzerland; b Kantonsspital Aarau, Clinic for Children and Teenagers, Division of Neuropediatrics, Aarau, Switzerland; c St. Elisabethen Krankenhaus Lörrach, Clinic of Childhood and Adolescent Psychiatry and Psychotherapy, Lörrach, Germany; d University of Basel, University Children's Hospital, Division of Neuropediatrics and Developmental Medicine, Basel, Switzerland

**Keywords:** ADHD, Attention deficit hyperactivity disorder, BESA, Brain Electric Source Analysis, BMI, Body mass index, CDA, Contralateral delay activity, CON, Waiting-list control group, IDS-2, Intelligence and Development Scales-2, EEG, Electroencephalogram, ERPs, Event-related potentials, HEOG, Horizontal eletrooculogram, JTG, Judo training group, MABC-2, Movement Assessment Battery for Children-2, RPE, Rating of perceived exertion, VEOG, Vertical eletrooculogram, Change detection task, Contralateral delay activity, Exercise, Martial arts, Executive function

## Abstract

**Background:**

Children with Attention Deficit Hyperactivity Disorder (ADHD) face deficits in working memory capacity that often persist into adulthood. In healthy peers, exercise targeting motor skill acquisition benefits visuospatial working memory, but its potential to reduce ADHD-related deficits remains unclear. We investigated the effect of a judo training program targeting motor skills on behavioral and neurocognitive indices of working memory capacity in children with ADHD.

**Methods:**

Children with ADHD aged 8 to 12 years (*N* = 57) were randomly allocated to a judo training group and a wait-list control group. The training program encompassed 120 min of judo per week over three months. Before and after the intervention period, participants completed a bilateral Change Detection task with low and high memory load conditions and the Movement Assessment Battery for Children-2 (MABC-2). The contralateral delay activity (CDA) elicited by the cognitive task was recorded using electroencephalography.

**Results:**

Compared to the control group, the judo training group showed a higher K-score on the Change Detection task and an increased negativity of the CDA on the high load condition following the intervention, when pretest scores (and confounders) were accounted for. In contrast, no group differences were found for MABC-2 score.

**Conclusion:**

In children with ADHD, judo training may complement the pharmacological treatment by increasing the effectiveness of working memory maintenance processes. On a behavioral level, this improvement is accompanied with an increased capacity to store visuospatial information.

## Highlights

• Judo training increased visuospatial working memory capacity in children with ADHD.
• Increased contralateral delay activity at high memory load was found following judo training.
• This change indexed more effective maintenance processes and/or increased attentional control.
• Benefits for working memory capacity occurred despite no changes in motor skills.

## 1 Introduction

The global prevalence of Attention Deficit Hyperactivity Disorder (ADHD) is about 5 % and an increasing trend over time has been reported among preadolescents (Sayal et al., 2018). Developmentally-inappropriate patterns of inattention, hyperactivity and/or impulsivity (in at least two settings) with an onset before children turn 12 years form its core symptoms (Association, 2013). Children with ADHD further face difficulties in executive function (Landis et al., 2021), with working memory problems in particular predicting long-term outcomes, such as symptom severity (van Lieshout et al., 2017). Moreover, impaired maintenance and manipulation of information in working memory is shared by the majority of pediatric patients, even though cognitive profiles related to ADHD are characterized by heterogeneity (Kofler et al., 2019). Poor performance on working memory tasks has been associated with an increased risk for grade retention and placement in special classes as well as problems with organizational skills, reading comprehension and mathematics (Fried et al., 2016, Kofler et al., 2018). Comparing different modalities of working memory, the ability to maintain visuospatial information in particular predicted long-term academic achievement (Sarver et al., 2012).

The proportion of children with ADHD receiving pharmacotherapy is increasing globally (Raman et al., 2018), but treatment with psychostimulants has only transient effects that do not normalize cognitive deficits (Tamminga et al., 2021). The international consensus supports the use behavioral strategies as first-line treatment in children with light to moderate ADHD symptoms (Seixas et al., 2012). Among them, structured exercise has the potential to support the treatment of ADHD as it acts on mechanisms that are impaired across several neurodevelopmental disorders (Ludyga et al., 2021). This is supported by *meta*-analytical evidence suggesting moderate benefits of exercise on executive function in children with ADHD, but it should be noted that the effect sizes were obtained from tasks assessing components other than working memory (Liang et al., 2021). The few experimental findings available on the effects of exercise on working memory showed inconsistent results. While a program that focused on game-based aerobic activities did not improve this cognitive function (Bustamante et al., 2016), both unspecific exercise and exercise aimed at promoting specific motor skills elicited benefits for working memory (Ziereis and Jansen, 2015). The exercise type may be a key to changes in working memory, given that *meta*-analytical findings from healthy populations have found more pronounced improvements across cognitive domains following exercise types that challenge body coordination (Ludyga et al., 2020). Based on a recent neurodevelopmental model, the effectiveness of such exercise types is due to an interrelation of working memory and motor skill acquisition (Ludyga et al., 2022). The model posits that children engaging in exercise demanding complex motor skills may be able to compensate for immature working memory functions by recruiting additional motor system support. Martial arts in particular are characterized by such demands, given that many techniques and technical actions in this sports type rely on body coordination and related motor skills (Eganov et al., 2020). In judo, for example, this focus is underlined by the observation that the number of different techniques and their variable use predict the success in a competition (Franchini et al., 2008), whereas physical fitness does not allow the discrimination of competitive rankings (Franchini et al., 2011). An influence of martial arts on working memory is further indicated by the neurocognitive profile of athletes experienced with this sports type. Children with regular engagement in martial arts for more than two years showed a higher working memory capacity than both sedentary peers (Alesi et al., 2014) and children practicing team sports for a similar time span (Giordano et al., 2021). Neuroimaging findings further revealed that children practicing judo are characterized by increased gray matter volume and white matter connectivity across different functional units (e.g. fronto-parietal network, corpus callosum) (Toh et al., 2018, Jacini et al., 2009), which are associated with working memory capacity (Bathelt et al., 2018, Darki and Klingberg, 2015). Judo therefore has much potential to elicit benefits for this cognitive ability and at the same time, it is characterized by the lowest injury risk in children when compared to other martial arts (Yard et al., 2007).

Recalling the characteristics of judo, it is expected to benefit visuospatial working memory as this storage system has been associated with motor sequence learning and early adaptation to changing task demands (Seidler et al., 2012). Visuospatial working memory can be assessed with a Change Detection task, which probes a bi-laterally presented memory array with a low or high number of items (Delvenne et al., 2011). When combined with electroencephalographic recordings, insights into subtle cognitive processes can be gained from the contralateral delay activity (CDA) elicited by the memory array (Downes et al., 2017). The CDA is a negative slow wave with highest amplitudes distributed across the parieto-occiptal region. Its amplitude increases with set size and relies on the difference between the attended and non-attended hemifield (Luria et al., 2016). As a high negativity of the CDA is evident in individuals with high working memory capacity, the diminished amplitudes in ADHD patients at adult age indicates that the developmental lag may extend beyond adolescence (Wiegand et al., 2016). Even though the CDA allows insights into processes underlying changes in behavioral performance on the Change Detection task, it has not yet been applied to study treatment effects in children with ADHD.

We aimed to investigate the effects of judo training program on behavioral and neurocognitive indices of visuospatial working memory capacity in children with ADHD. Based on the evidence indicating cognitive benefits of exercise with coordinative demands, we expected a higher the number of items that could be recalled correctly on the Change Detection task along with an increased negativity of the CDA in the judo training group (JTG) compared to a wait-list control group (CON) following the intervention period.

## 2 Methods

### 2.1 Participants

Children with ADHD were directly recruited from local clinics in Basel (Switzerland) and Lörrach (Germany) between 2019 and 2021. Sample size was calculated a priori using G*Power 3.1.9.4 (Faul et al., 2007). A *meta*-analysis including three studies, which investigated possible benefits of predominantly aerobic exercise for executive function in children with ADHD, detected a moderate positive effect (Cerrillo-Urbina et al., 2015). Effect sizes were not available for martial arts training in children with ADHD, but a previous study in healthy peers found large improvements for executive function following 12 weeks of judo training (Ludyga et al., 2021). Given that individuals with lower baseline cognition benefit more from exercise (Ishihara et al., 2020), a similar or even larger effect of judo training was expected for children with ADHD. Based on an alpha level of *p* = 0.05, the initial power analysis (ANCOVA corrected for baseline scores) indicated that 56 participants (28 per group) were required to reach 85 % statistical power. The study was stopped, when the target sample size was achieved.

Participants had to express their willingness to take part in the study and written informed consent was obtained from their legal guardians. Eligible participants were right-handed children aged 8 to 12 years with an ADHD diagnosis according to the DSM-5^2^ and corrected-to or normal vision. Additionally, only participants undergoing pharmacotherapy with methylphenidate or dexamphetamine for at least three months prior to the intervention were included to reduce inter-individual variations in symptom severity. Regular participation (once a week) in martial arts three months prior to the intervention, dosage changes methylphenidate/dexamphetamine within one month prior to the intervention, additional therapy forms, Autism Spectrum Disorder and/ or structural epilepsy as comorbid conditions, and injuries or diseases affecting the functionality of the right hand as well as any chronic disease classified as contraindication for exercise were defined as exclusion criteria. Prior to the study, the protocol was preregistered at the German Registry of Clinical Trials (DRKS00020125) and approved by the local ethics committee (Ethikkommission Nordwest- und Zentralschweiz). The guidelines set forth in the Declaration of Helsinki and its amendments were followed.

### 2.2 Study design

Using concealed allocation, participants were randomly assigned (stratum: low and high dosage of methylphenidate/dexamphetamine) to JTG and CON in a 1:1 ratio. The randomization list was computer-generated using sealed envelope^TM^ (London, UK), included variable block sizes of 4 and 6, two groups and low and high dose of psychostimulants as stratum. Allocation was concealed using sequentially numbered opaque envelopes. After the first lab visit, the principal investigator informed participants on their group allocation via telephone and/ or email. Information on the allocation was not shared with lab personnel performing the outcome assessments.

Before and after the intervention period, participants performed a computerized Change Detection task and the Movement Assessment Battery for Children-2 (MABC-2). During the cognitive task, ERPs were recorded via EEG. Additionally, participants’ body mass index (BMI) was assessed and the Family Affluence Scale (Currie et al., 2008), Intelligence and Development Scales-2 (IQ screening items) (Grob and Hagmann von Arx, 2018) and Pubertal Development Scale (Petersen et al., 1988) were administered. Their legal guardians were asked to fill in the Conners-3 Scales (Lidzba et al.). Pre- and posttests were scheduled during the same time of the day. Participants were instructed to have their last meal two hours before the assessments and to refrain from the engagement in sports activities on prior to the laboratory visit. Participants completed measurements in a calm environment and at a room temperature of 21 to 22°.

### 2.3 Intervention

The judo training program consisted of two weekly 60-min sessions over a period of 12 weeks. Participants were trained in a group setting and supervised by one or two instructors (based on group size). The aim of the training was to prepare judo beginners for their first belt examination by teaching basic judo techniques for attack, defense and injury prevention. While the judo sessions mainly focused on learning and refining techniques, they also included playful and children-appropriate exercises that targeted physical fitness. Both components were practiced together in Randori. Participants were asked to rate perceived exertion (RPE) immediately following judo practice on four of the 24 scheduled sessions.

### 2.4 Cognitive task

**Fig. 1:**
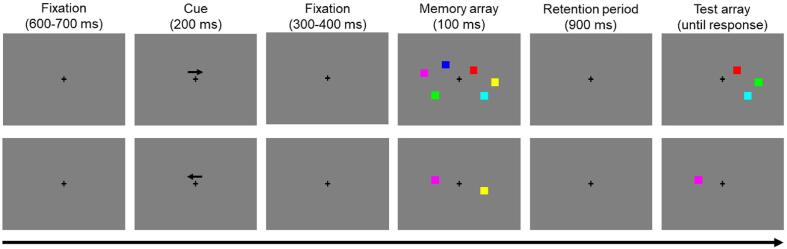
Example high load (upper row) and low load (lower row) trials of the Change Detection paradigm.

An existing script of a Change Detection paradigm (Delvenne et al., 2011) was modified and administered with E-Prime 3.0 (PST, USA). The task required participants to encode a memory set containing an array of coloured squares (Fig. 1). Following the presentation of a fixation cross (600 to 700 ms), a cue (200 ms) indicated whether the colored squares of either the right or left hemi-field had to be remembered. The memory array (100 ms) was preceded by an inter-stimulus interval (300 to 400 ms) and followed by a retention period (900 ms) during which the screen went blank. The memory array included either 1 or 3 differently colored squares on each hemifield. Trials with low and high memory load were presented with equal probability and their sequence was randomized. The position of the squares varied randomly on the y-axis (within a pre-specified range), but were fixed in their distance to the center based on the x-axis. The test array contained the square(s) from the cued hemifield with or without a change in color. At this stage, participants had to indicate whether or not the test array is different from the memory array by pressing one of two buttons on a serial response box. The task included a practice block with 16 trials, followed by four blocks with 56 trials each. The test blocks were interspersed by short breaks to have participants relax their eyes. Reaction time on response-correct trials and accuracy were extracted for each set size. In addition, K-score was calculated using the following formula: set size * ((hit rate – false alarm rate) / (1 – false alarm rate)) (Rouder et al., 2011).

### 2.5 EEG recording and processing

The electroencephalogram was recorded from 64 active electrodes during the Change Detection task. Their position corresponded to the 10:10 system, with Cz used as online reference and AFz as ground. Prior to the recordings, impedance was reduced to 10 KΩ or lower (on at least 90 %) by filling electrodes embedded in a flexible head cap with a highly conductive gel. Using the amplifier ActiCHamp (BrainProducts, Germany), data was band-pass filtered (0.01 to 100 Hz) and digitized with a sampling rate of 500 Hz. Collected data was submitted to BESA Research 7.1 (Brain Electric Source Analysis, Germany) for offline processing. Blinks were detected by virtual HEOG and VEOG channels and then corrected with automatic adaptive artefact correction. This approach applies principal component analysis on continuous EEG data to remove artefact components from brain signals. Following high-pass filtering (forward phase shift of 0.1 Hz; slope 6 dB/octave) and baseline correction (−200 ms to stimulus onset), artifacts surviving the correction procedure were rejected based on individual gradient (*M* = 75 µV, *SD* = 0.5 µV) and amplitude thresholds (*M* = 117.5 µV, *SD* = 19.9 µV). Subsequently, segments were built for a latency range from 0 to 900 ms following the onset of the memory array and averaged separately for trials with low and high working memory load (Table S1, Appendix). The grand averaged segments were submitted to low-pass filtering (zero-phase shift of 30 Hz; slope 24 dB/octave) and the reference was changed to the average of all channels. The CDA was derived by subtracting contralateral from ipsilateral waveforms separately for trials cueing the left and trials cueing the right hemifield. Subsequently, the waveforms for both cue types were averaged. The selection of latency ranges and regions of interest for deriving the ERP components was informed by findings showing a parieto-occiptal distribution of the CDA within 300 to 900 ms relative to the onset of the memory array. As previous research focused on adults mainly, the latency range was adjusted slightly based on a preliminary inspection of the ERP waves. For statistical analysis, the CDA amplitude was extracted as the mean over the 250 to 600 ms latency range relative to the onset of the memory array using an average of the following electrodes: *P*3/4, P7/P8, PO3/PO4, PO7/PO8.

### 2.6 Assessment of motor skills

Depending on the age of participants (≤11 y or ≥ 12 years), the appropriate version of the Movement Assessment Battery for children – 2 (MABC-2) (Bös and Kastner, 2015) was administered. This battery differentiates manual dexterity (3 items), balance skills (3 items) and aiming/catching (2 items). The MABC-2 is sensitive to developmental changes and shows a high reliability as well as factorial validity. Based on the raw scores of each dimension, age and gender-corrected standard scores were derived and combined to yield a standard score for the overall motor skills.

### 2.7 Statistical analyses

The Shapiro Wilk and Levene’s tests were employed to check whether collected data fulfilled specific criteria for the application of analysis of variance (normal distribution and homogeneity of variances). Pearson correlation or point-biserial correlations were calculated between potential confounders (age, sex, body mass index, pubertal status, and socio-economic status) and outcomes (K-score, CDA). Variables showing a correlation of at least r = 0.2 or a significant correlation with one or more outcomes served as covariates in subsequent main analyses. Our method used for hypothesis testing was informed by a simulation study that recommended the use of an ANCOVA approach to analyze treatment effects on continuous outcomes, especially when baseline imbalances can be expected (Egbewale et al., 2014). In case of homogenous repeated measures covariance, this statistical method is even preferred over a repeated measures alternative (Winkens et al., 2007). Based on preliminary examination of covarainces with Box’s M test, this criterion was fulfilled for all outcomes. Consequently, we examined the effects of the judo training program on working memory indices and motor skills by using a series of ANCOVAs testing between-groups differences in posttest scores (K-score, CDA amplitude, MABC-2 score) while controlling for pretest scores and potential confounders, if necessary. Using path-analysis, we further examined the correlation between posttest K-score and posttest CDA amplitude, while controlling for autoregressive effects (pretest scores), the treatment group variable and potential confounders. Student’s T-tests were used to test whether coefficients differed from zero. In ANCOVA and path-analysis, the level of statistical significance was set to *p* < 0.05. Effect sizes were considered small, medium and large at η^2^ ≥ 0.01 or r ≥ 0.20, η^2^ ≥ 0.06 or r ≥ 0.30, and η^2^ ≥ 0.14 or r ≥ 0.40, respectively.

## 3 Results

**Fig. 2:**
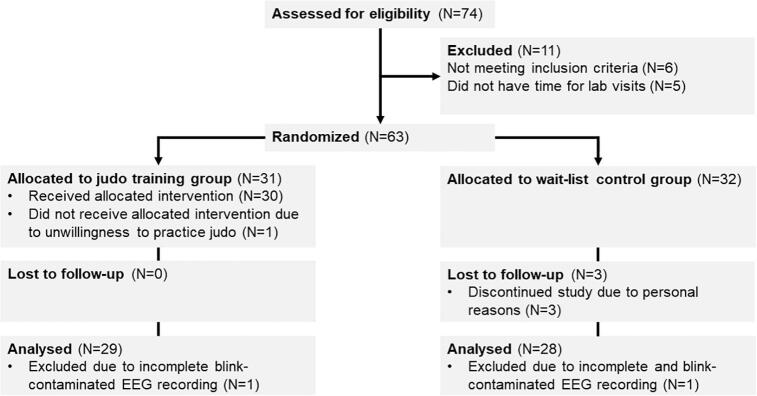
Flowchart of participants.

Fifty-seven participants completed the study (Fig. 2). Their psychopathology, intelligence, pubertal status, socioeceonomic status and anthropometrics are shown in Table 1. The compliance among the JTG was 81.9 % (*SD* = 10.9) and an average RPE of 13.3 (*SD* = 0.7) indicated moderate intensity. Uncorrected means of both groups’ pre- and posttest outcomes are shown in Table 2. Based on preliminary correlational analyses, outcomes had to be accounted for age, BMI and/ or socio-economic status (Table S2, Appendix).

**Table 1:** Characteristics of participants in the wait-list control group and judo training group.

	Wait-list control group (*N* = 18 m / 10f)		Judo training group (*N* = 23 m / 6f)	
	*M*	*SD*	*M*	*SD*
Conners 3 - ADHD Index (*t*-value)	64.1	5.6	62.7	6.2
Conners 3 - Global Index (*t*-value)	65.9	6.6	65.1	6.6
MPH/DEX dose in mg^.^d^−1^	30.3	12.2	26.7	13.6
Age in years	10.8	1.2	10.0	1.2
Body weight in kg	38.7	8.8	38.6	14.0
Body mass index in kg^.^m^−2^	18.3	2.6	18.2	4.2
Family Affluence Scale	5.9	1.5	5.6	1.4
IDS-2 IQ screening (value points)	9.7	3.8	12.4	5.3
Pubertal Development Scale	1.3	0.6	1.3	0.7

*Notes:* The Conners 3 Scales ask parents to evaluate the behavior of their child in the last month. In some participants, values may indicate subclinical ADHD due to their treatment with psychostimulants. Missing values on IDS-2 IQ screening (*N* = 2) and Pubertal Development Scale (*N* = 6) were estimated with multiple imputation. DEX = Dexamphetamine; IDS-2 = Intelligence and Development Scales-2 (IQ screening items: matrices and naming of categories); MPH = Methylphenidate.

**Table 2:** Cognitive performance, contralateral delay activity and motor skills of wait-list control group and judo training group at pre- and posttest.

	Wait-list control group (*N* = 18 m / 10f)				Judo training group (*N* = 23 m / 6f)			
	Pretest		Posttest		Pretest		Posttest	
	*M*	*SD*	*M*	*SD*	*M*	*SD*	*M*	*SD*
Reaction time in ms	1053.0	254.2	954.1	261.2	1211.0	296.2	1057.4	264.5
K-score	1.9	0.7	1.9	0.9	1.7	0.5	2.0	0.5
CDA low load in µV	1.0	2.9	−0.1	2.5	−0.2	2.5	−0.8	1.9
CDA high load in µV	−1.9	3.3	−1.3	2.2	−1.7	2.1	−2.7	2.5
MABC-2 score	32.0	21.1	45.6	28.4	29.7	22.3	38.1	26.9

*Notes:* CDA = Contralateral delay activity; MABC-2 = Movement Assessment Battery for Children-2.

Comparing behavioral performance, the JTG (*M* = 2.13; *SE* = 0.11) had higher K-Score following the intervention than the CON (*M* = 1.77; *SE* = 0.11), when controlled for age and K-score at pretest, *F*(1, 53) = 5.00, *p* = 0.030, η^2^ = 0.086. There was no indication of a speed-accuracy trade-off as reaction time at post-test did not differ between groups, after accounting for pretest reaction time, age and socio-economic status, *F*(1, 52) = 0.45, *p* = 0.508, η^2^ = 0.008.

**Fig. 3:**
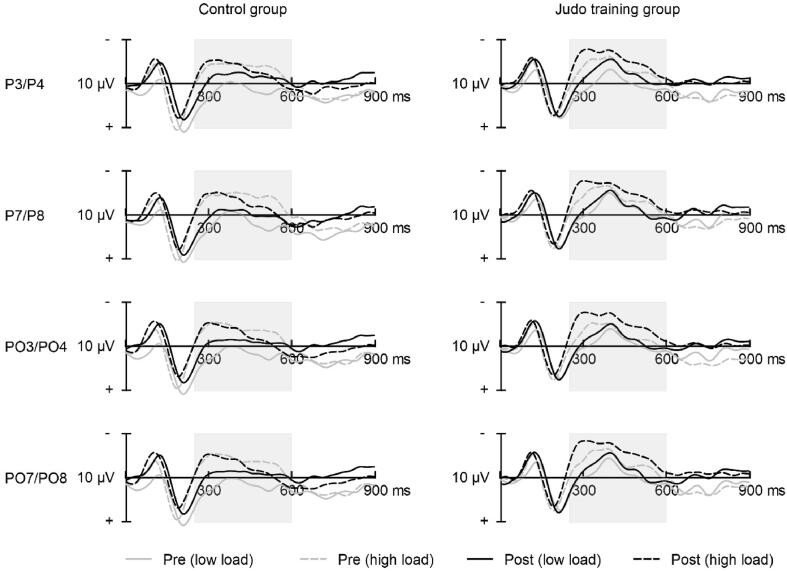
Pre- and posttest CDA waveforms (contralateral – ipsilateral) elicited by the low and high working memory load conditions of the Change Detection task displayed for the control group and judo training group. *Notes:* The latency range marked by the vertical grey bar was used to calculate the CDA amplitude.

The CDA waveforms of both groups are displayed in Fig. 3. The JTG (*M* = −2.65 µV; *SE* = 0.43) showed a higher negativity of the posttest CDA amplitude in response to the high load condition than the CON (*M* = −1.37 µV; *SE* = 0.44), when CDA amplitude at pretest and socioeconomic status were accounted for, *F*(1, 53) = 4.30, *p* = 0.043, η^2^ = 0.075. In contrast, the CDA amplitude elicited by the low load condition did not differ between groups in the corrected analysis, *F*(1, 54) = 0.03, *p* = 0.858, η^2^ = 0.001.

Based on path-analysis, there was a moderate correlation between posttest CDA amplitude in response to the high load condition and K-Score at posttest, *r* = 0.31, *p* = 0.028, when autoregressive effects, the treatment group, socio-economic status and age were accounted for. In contrast, no such correlation was found for the low load condition in the corrected analysis, *r* = 0.11, *p* = 0.396.

With regard to motor skills, MABC-2 score did not differ between the JTG and the CON at posttest, when the ANCOVA included pretest scores, socioeconomic status and BMI as covariates, *F*(1, 51) = 2.23, *p* = 0.328, η^2^ = 0.019.

## 4 Discussion

The JTG had a higher K-score following the intervention period than the CON, whereas no group differences were found in reaction time. This indicates that at comparable speed, the judo training enhanced the number of items that could be maintained in visuospatial working memory. The increase in K-score was accompanied by an increased negativity of the CDA amplitude in the high load condition of the Change Detection task.

Recalling the cognitive endotypes of ADHD, impairments in working memory seem to be most consistent characteristic across participants (Kofler et al., 2018). These impairments can be caused at different stages of working memory operations and may originate from difficulties in the maintenance or manipulation of items. The version of the Change Detection task employed in our study mainly demands the storage of visuospatial information. Consequently, an increased K-score in the JTG reflects improvement in the capacity to maintain items from a memory array. This may also translate into benefits for more global working memory performance, given that the storage of information is a prerequisite for their subsequent manipulation. Our results are in line with a review indicating that exercise interventions in children with a neurodevelopmental disorders, such as ADHD, improve impaired cognitive functions, suggesting a normalization tendency (Ludyga et al., 2021). Whereas the majority of previous experimental studies focused on inhibitory control and cognitive flexibility, limited evidence also showed improvements of working memory after 12 weeks of engagement in skill-based or unfocused exercise (Ziereis and Jansen, 2015). Both exercise programs showed a trend towards increased motor skills as assessed with the MABC-2, lending support to the neurodevelopmental model that such benefits may translate into improved performance on challenging working memory tasks (Ludyga et al., 2022). In contrast, the judo training did not lead to group differences in overall performance on MABC-2 as the JTG and the CON showed similar improvements from pre- to posttest. This lack of group differences was not expected as engagement in judo is known to elicit benefits for motor skills (Fukuda et al., 2013). Consequently, the effects of judo training on working memory capacity may not depend on changes in fine and gross motor skills assessed with the MABC-2 or these cognitive effects occur even before differences between groups can be detected.

On a neurocognitive level, insights into working memory processes sensitive to judo training are provided by the CDA. Following the intervention period, a higher negativity of this component was found in the JTG compared to the CON, but only in the high load condition. Consequently, judo training seems to promote the effective maintenance of several items in visuospatial memory rather than the efficient storage of a single item. This change cannot be explained by altered sensory processing, given that a potential confounding effect is controlled by calculating the CDA from the difference in slow wave activity between the attended and non-attended hemifield. However, there are still different mechanisms that may have contributed to the observed group difference. In a previous experiment, CDA predicted inter-individual differences in a variety of cognitive abilities via both working memory capacity and attentional control (Unsworth et al., 2015). In this context, capacity describes the maintenance of multiple items in an active state, whereas attentional control encompasses the protection of these items, the selection of target representations for active maintenance, and the restriction of access of irrelevant or distracting information to working memory. The processes indexed by the CDA seem to respond to direct demands on capacity as training of the working memory span yielded an increase of its negativity (Zhang et al., 2020). We found a similar effect following judo practice, which may in part be due to the working memory demands in the early phase of training. In general, the initial acquisition of motor skills requires the maintenance of task-relevant information, e.g. instructions, specific task demands and movement sequences (Furley and Wood, 2016). Visuospatial working memory in particular is required when movements need to be combined to sequences and when acquired motor skills are adapted to a changing environment (Seidler et al., 2012). Our judo intervention constantly challenged this working memory function as trainers taught multiple falling, throwing and grappling techniques over the intervention period. All techniques had to be combined in an unpredictable environment during “Randori”. This free fighting practice required the maintenance of visuospatial cues (e.g. joint positions, movement patterns) to anticipate and react to the opponent’s actions while standing or on the ground. Randori is the training modality that induces demands that are most closely related to those of an actual judo match (Franchini et al., 2014). The use of multiple techniques and their variation predicts success in judo competition (Franchini et al., 2008), suggesting that the development of cognitive abilities rather than physical fitness promotes the advancement of the competitive level in judo. Recalling that motor sequence learning and early adaptation of motor sequences rely on visuospatial memory (Seidler et al., 2012), the acquisition of multiple basic judo techniques may have triggered the enhancement of working memory capacity. This is indirectly supported by the observation that the judo training increased the CDA negativity at high load, while MABC-2 overall score did not develop differently over the intervention period between groups.

Despite the use of an experimental design and both behavioral and neurocognitive indices of working memory capacity, some limitations should be noted when interpreting the results. First, the sex distribution was not equal between groups and may have affected the outcomes as sex has been suggested to moderate exercise-induced effects for cognitive performance (Ludyga et al., 2020). However, our preliminary analysis showed that sex was not related to K-score and CDA amplitudes following the intervention, suggesting that its moderating role does not necessarily apply to children with ADHD or children with a prepubertal status (as indicated by the Pubertal Development Scale). Second, the present findings may not generalize to all children with ADHD as we only included those undergoing treatment with psychostimulants. This approach was chosen to limit variations in symptom severity, given that treatment guidelines only recommend the use of stimulants in children affected by severe ADHD symptoms (Seixas et al., 2012). The continuation of pharmacological treatment over the intervention period further limits conclusions to the effectiveness of judo as an additional treatment component. However, an experimental study found that in children with ADHD, the acute cognitive response to a single exercise session did not differ between users and non-users of methylphenidate (Medina et al., 2010). This is a first indication that the benefits of judo may not depend on the pharmacological treatment, but futures studies need to verify the lack of an influence of the treatment on long-term benefits for working memory capacity. Third, the treatment effect might be biased by participants’ expectations due to the selection of a wait-list control group rather than an active control group. However, active control conditions have not been considered more stringent than the use of a wait-list in clinical trials with behavioral interventions (Freedland et al., 2011). This is due to the opportunity to engage in non-study behaviors that influence the outcome, but do not dependent on a clinical provider. Additionally, a *meta*-analysis investigating potential moderators of the cognitive effects of exercise across all ages revealed no influence of the choice of the control group (active vs passive) on attention, executive function, and memory (Ludyga et al., 2020). Fourth, the lack of group differences in motor skills following the intervention period might be due the choice of the MABC-2 battery. This set of tasks assesses fine and gross motor skills, so that improvements in more judo-specific skills might be masked. Lastly, it remains unclear whether judo training had a general or specific effect on working memory as we focused on the capacity to store visuospatial information.

## 5 Conclusion

The prescription of a 12-week judo training to children with ADHD promises improvements in working memory capacity. These beneficial effects can be observed on both behavioral and neurocognitive level. Judo training increases the number of items that can be stored in visuospatial working memory and promotes the effectiveness of associated maintenance processes indexed by the CDA. As abnormalities in the CDA reflect an ADHD-related developmental lag that often persists into adulthood, judo has the potential to support conventional treatment approaches.

## Funding

This work was supported by the Swiss National Science 10.13039/100016163Foundation [32003B_188488].

## CRediT authorship contribution statement

**Sebastian Ludyga:** Conceptualization, Data curation, Formal analysis, Funding acquisition, Investigation, Methodology, Project administration, Resources, Software, Supervision, Visualization, Writing – original draft. **Manuel Mücke:** Investigation, Methodology, Writing – review & editing. **Rahel Leuenberger:** Investigation, Project administration, Writing – review & editing. **Fabienne Bruggisser:** Investigation, Project administration, Writing – review & editing. **Uwe Pühse:** Conceptualization, Resources, Writing – review & editing. **Markus Gerber:** Conceptualization, Resources, Writing – review & editing. **Andrea Capone-Mori:** Project administration, Writing – review & editing. **Clemens Keutler:** Project administration, Writing – review & editing. **Mark Brotzmann:** Project administration, Writing – review & editing. **Peter Weber:** Project administration, Conceptualization, Writing – review & editing.

## Declaration of Competing Interest

The authors declare that they have no known competing financial interests or personal relationships that could have appeared to influence the work reported in this paper.

## Figures and Tables

**Table S1:** The number of segments contributing to the contralateral delay activity in the low load and high load conditions displayed for the wait-list control group and the judo training group at pre- and posttest.

	Wait-list control group				Judo training group			
	Pretest		Posttest		Pretest		Posttest	
	*M*	*SD*	*M*	*SD*	*M*	*SD*	*M*	*SD*
Low load	86.0	16.2	84.4	20.8	82.7	18.6	86.8	16.0
High load	73.4	15.9	70.9	21.2	68.1	15.9	76.0	15.1

*Notes:* Only response-correct segments were averaged. The total number of good segments therefore depends on the signal-to-noise ratio and the accuracy on the Change detection task.

**Table S2:** Correlations between potential confounders at pretest and outcomes at posttest.

	Reaction time	K-score	CDA (low load)	CDA (high load)	MABC-2
Age	−0.57*	0.22	−0.10	−0.12	0.19
Sex	0.17	0.17	−0.13	−0.12	−0.10
Body mass index	0.03	0.07	0.04	0.10	−0.25
Pubertal Development Scale	−0.11	0.15	−0.17	−0.13	−0.10
Family Affluence Scale	−0.15	−0.14	0.18	0.28*	0.21
IDS-2 IQ screening	0.03	0.05	0.08	0.06	−0.14

*Notes:* *p < 0.05; CDA = Contralateral delay activity; IDS-2 = Intelligence and Development Scales-2 (IQ screening items: matrices and naming of categories); MABC-2 = Movement Assessment Battery for Children-2.

## Data Availability

Data will be made available on request. Additionally, data from the JETPAC study will be made available on SWISSUbase within 12 months.
